# Functional Principal Component Analysis and Randomized Sparse Clustering Algorithm for Medical Image Analysis

**DOI:** 10.1371/journal.pone.0132945

**Published:** 2015-07-21

**Authors:** Nan Lin, Junhai Jiang, Shicheng Guo, Momiao Xiong

**Affiliations:** 1 Human Genetics Center, Department of Biostatistics, School of Public Health, the University of Texas Health Science Center at Houston, Houston, Texas, 77030, United States of America; 2 State Key Laboratory of Genetic Engineering and Ministry of Education Key Laboratory of Contemporary Anthropology, School of Life Sciences and Institutes of Biomedical Sciences, Fudan University, Shanghai, 200433, China; Pennsylvania State University, UNITED STATES

## Abstract

Due to the advancement in sensor technology, the growing large medical image data have the ability to visualize the anatomical changes in biological tissues. As a consequence, the medical images have the potential to enhance the diagnosis of disease, the prediction of clinical outcomes and the characterization of disease progression. But in the meantime, the growing data dimensions pose great methodological and computational challenges for the representation and selection of features in image cluster analysis. To address these challenges, we first extend the functional principal component analysis (FPCA) from one dimension to two dimensions to fully capture the space variation of image the signals. The image signals contain a large number of redundant features which provide no additional information for clustering analysis. The widely used methods for removing the irrelevant features are sparse clustering algorithms using a lasso-type penalty to select the features. However, the accuracy of clustering using a lasso-type penalty depends on the selection of the penalty parameters and the threshold value. In practice, they are difficult to determine. Recently, randomized algorithms have received a great deal of attentions in big data analysis. This paper presents a randomized algorithm for accurate feature selection in image clustering analysis. The proposed method is applied to both the liver and kidney cancer histology image data from the TCGA database. The results demonstrate that the randomized feature selection method coupled with functional principal component analysis substantially outperforms the current sparse clustering algorithms in image cluster analysis.

## Introduction

Image clustering is to cluster the objects into groups such that the objects within the same group are similar, while the objects in different groups are dissimilar [1, 2]. Image clustering is a powerful tool to better organize and represent the images in image annotation, image indexing, image segmentation and subtype disease identification. Dimension reduction of the original images is an essential to the success of the image clustering analysis.

Feature extraction and feature selection are two popular methods for dimension reduction. A widely used method for feature extraction is the principal component analysis (PCA). However, PCA does not explore the spatial information within the image. It takes a set of spectral images as an unordered set of high dimensional pixels [3]. Spatial information is an important component for image cluster and classification analysis. To overcome the limitations of PCA and to utilize spatial information of the image signals, the functional expansion of the images based on Fourier and wavelet transform are proposed as a useful tool for image feature extraction and data denoising [4]. Recently, wavelet PCA which is based on the principal components for a set of wavelet coefficients is proposed [3] to explore both the spatial and the spectral information. The wavelet PCA improves the efficiency of image feature extractions, but does not explicitly consider smoothing image signals over space. To overcome this limitation and fully utilize both the spatial and spectral information, we extend one dimensional functional principal component analysis (FPCA) to high dimensional FPCA.

Traditional statistical methods for image clustering and classification analysis often fail to obtain accurate results because of the high dimensional nature of the images [5]. Noisy and irrelevant features might result in over-fitting. The high dimensionality reduces the time efficiency of the clustering algorithms [6]. As a result, the high dimensionality of images provides a considerable challenge for designing efficient clustering algorithms [6]. Removing noisy, redundant and irrelevant features while retaining a minimal feature subset will dramatically improves the accuracy of image cluster analysis [7]. The sparse algorithm is a widely used method for feature selection in which a lasso-type penalty provides a general framework to simultaneously find the clusters and the important clustering features in image cluster analysis [8, 9]. Although the sparse clustering methods can improve the accuracy, it may fail to generate reasonable clusters when the data include a few outliers. In practice, the performance of sparse clustering depends on the selection of penalty parameters and threshold for the cutting off features. However, the selections of penalty parameters and the threshold have been proved to be difficult.

Alternatively, a randomized method is proved to be useful when the number of features is prohibitively large [10]. An efficient randomized feature selection method for *k*-means clustering randomly selects the features with probabilities that are calculated via singular value decomposition of the data matrix [6, 11]. This algorithm has a very useful property that can theoretically guarantee the quality of the clusters. To the best of our knowledge, this efficient and provable accurate randomized feature selection algorithm has not been applied to the image cluster analysis.

Although feature selection and feature extraction are widely used to reduce the dimensionality of the image, we have observed very few practices to combine feature selection and feature extraction together for dimension reduction. We can expect that applying feature selection algorithm to select extracted features from a set of artificial features that are computed via feature extraction will improve the accuracy of image clustering.

The purpose of this paper is to develop a comprehensive sparse clustering algorithm with four components for image cluster analysis. The first component is to use high dimensional FPCA as a feature extraction technique. The second component includes a theoretically provable accurate randomized feature selection algorithm. The third component is to combine feature selection and feature extraction together for dimensionality reduction. The fourth component is spectral clustering with low rank matrix decomposition that can effectively remove noises and ensure the robustness of the algorithms. To evaluate its performance for image cluster analysis, the proposed method is applied to 176 ovarian cancer histology images with the drug response status (106 images with positive drug response and 70 images with drug resistance) and 188 kidney histology images (121 images from tumor samples and 67 images from normal samples) from the TCGA database. Our results strongly demonstrate that the proposed method for feature selection substantially outperforms other existing feature selection methods in the image clustering analysis. The R packages for implementing the proposed methods can be downloaded from our website http://www.sph.uth.tmc.edu/hgc/faculty/xiong/index.htm.

## Materials and Methods

### Two dimensional functional principal component analysis

One dimensional functional principal component analysis (FPCA) has been well developed [12]. Now we extend one dimensional FPCA to two dimensional FPCA. In a two dimensional region, *s* and *t* denote the coordinates in the *s* axis and *t* axis, respectively. Let *x*(*s*, *t*) be a centered image signal located at *s* and *t* of the region. The signal *x*(*s*, *t*) is a function of locations *s* and *t*.

A linear combination of functional values can be expressed as:

$$f=\int_{S}\int_{T}\beta (s,t)x(s,t)dsdt,$$ (1)

where β(*s*, *t*) is a weight function. To capture the variations in the random functions, we chose the weight function β(*s*, *t*) to maximize the variance of *f*. By the formula for the variance of stochastic integral [13], we have

$$\text{var}(f)=\int_{S}\int_{T}\int_{S}\int_{T}\beta (s_{1},t_{1})R(s_{1},t_{1},s_{2},t_{2})\beta (s_{2}.t_{2})ds_{1}dt_{1}ds_{2}t_{2},$$ (2)

where *R*(*s*_1_, *t*_1_, *s*_2_, *t*_2_) = cov(*x*(*s*_1_, *t*_1_), *x*(*s*_2_, *t*_2_)) is the covariance function of the image signal *x*(*s*, *t*). Since multiplying *β*(*s*, *t*) by a constant will not change the selected features, *Var*(*f*), we impose a constraint to make the solution unique:

$$\int_{S}\int_{T}\beta^{2}(s,t)dsdt=1$$ (3)

Therefore, to find the weight function, we seek to solve the following optimization problem:

$$\begin{array}{l} \text{max }\int_{S}\int_{T}\int_{S}\int_{T}\beta (s_{1},t_{1})R(s_{1},t_{1},s_{2},t_{2})\beta (s_{2},t_{2})ds_{1}dt_{1}ds_{2}t_{2}\\ \text{s.t. }\int_{S}\int_{T}\beta^{2}(s,t)dsdt=1. \end{array}$$ (4)

Using the Lagrange multiplier, we reformulate the constrained optimization problem Eq (4) into the following non-constrained optimization problem:

$$\underset{\beta }{\text{max }}\;\frac{\text{1}}{\text{2}}\int_{S}\int_{T}\int_{S}\int_{T}\beta (s_{1},t_{1})R(s_{1},t_{1},s_{2},t_{2})\beta (s_{2},t_{2})ds_{1}dt_{1}ds_{2}t_{2}+\frac{1}{2}\lambda (1-\int_{S}\int_{T}\beta^{2}(s_{1},t_{1})ds_{1}dt_{1}),$$ (5)

where *λ* is a penalty parameter.

By variation calculus [14], we define the functional

$$J[\beta ]=\frac{\text{1}}{\text{2}}\int_{S}\int_{T}\int_{S}\int_{T}\beta (s_{1},t_{1})R(s_{1},t_{1},s_{2},t_{2})\beta (s_{2},t_{2})ds_{1}dt_{1}ds_{2}t_{2}+\frac{1}{2}\lambda (1-\int_{S}\int_{T}\beta^{2}(s_{1},t_{1})ds_{1}dt_{1}).$$ (6)

Its first variation is given by

$$\begin{array}{l} \delta J[h]=\frac{d}{d\varepsilon }J[\beta (s,t)+\varepsilon h(s,t)]\\ \begin{array}{ccc} & & \;=\int_{S}\int_{T}[\int_{S}\int_{T} \end{array}[R(s_{1},t_{1},s_{2},t_{2})\beta (s_{2},t_{2})ds_{2}t_{2}-\lambda \beta (s_{1},t_{1})]h(s_{1},t_{1})ds_{1}dt_{1}=0. \end{array}$$ (7)

Since the expression above should be 0 at the maximizer for arbitrary *h*(*s*, *t*), and *h*(*s*, *t*) can be replaced by that certain form. Replacing *h*(*s*, *t*) by $\int_{S}\int_{T}R(s_{1},t_{1},s_{2},t_{2})\beta (s_{2},t_{2})ds_{2}t_{2}-\lambda \beta (s_{1},t_{1})$ in the above equation, we obtain

$$\delta J[h]=\int_{S}\int_{T}{\left[\int_{S}\int_{T}R(s_{1},t_{1},s_{2},t_{2})\beta (s_{2},t_{2})ds_{2}t_{2}-\lambda \beta (s_{1},t_{1})\right]}^{2}ds_{1}dt_{1}=0,$$ (8)

which implies the following integral equation

$$\;\int_{\text{S}}\int_{\text{T}}R(s_{1},t_{1},s_{2},t_{2})\beta (s_{2},t_{2})ds_{2}dt_{2}=\lambda \beta (s_{1},t_{1})$$ (9)

with an appropriate eigenvalue *λ*. The left side of the integral Eq (9) defines a two dimensional integral transform *R* of the weight function *β*. Therefore, the integral transform of the covariance function *R*(*s*_1_, *t*_1_, *s*_2_, *t*_2_)is referred to as the covariance operator *R*. The integral Eq (9) can be rewritten as

Rβ=λβ, (10)

where β(*s*, *t*) is an eigenfunction and referred to as a principal component function. Eq (10) is also referred to as a two dimensional eigenequation. Clearly, the eigenequation Eq (10) looks the same as the eigenequation for the multivariate PCA if the covariance operator and eigenfunction are replaced by the covariance matrix and eigenvector.

Since the number of the functional values is theoretically infinite, we may have an infinite number of eigenvalues. Provided the functions *X*_*i*_ and *Y*_*i*_ are not linearly dependent, there will be only *N*—1 nonzero eigenvalues, where *N* is the total number of sampled individuals (*N* = *n*_*X*_ + *n*_*Y*_), where *n*_*X*_ and *n*_*Y*_ are sample sizes for *X* and *Y*, respectively. The eigenfunctions satisfying the eigenequations are orthonormal [12]. In other words, Eq (10) generates a set of principal component functions

$$R\beta_{k}=\lambda_{k}\beta_{k},\text{ with }\lambda_{\text{1}}\geq \lambda_{\text{2}}\geq \cdots .$$ (11)

These principal component functions satisfy

1. $\int_{S}\int_{T}\beta_{k}^{2}(s,t)dsdt=1$ and
2. $\int_{S}\int_{T}\beta_{k}(s,t)\beta_{m}(s,t)dsdt=0\text{, for all }m<k.$

The principal component function *β*_1_ with the largest eigenvalue is referred to as the first principal component function and the principal component function *β*_2_ with the second largest eigenvalue is referred to as the second principal component function, etc.

### Computations for the principal component function and the principal component score

The eigenfunction is an integral function and difficult to solve in a closed form. A general strategy for solving the eigenfunction problem in Eq (9) is to convert the continuous eigen-analysis problem to an appropriate discrete eigen-analysis task [12]. In this paper, we use basis function expansion methods to achieve this conversion.

Let {*ϕ*_*j*_(*t*)}bea series of Fourier functions. We expand each image signal function *x*_*i*_(*s*, *t*) as a linear combination of the basis function *ϕ*_*j*_:

$$x_{i}(s,t)=\sum_{k=1}^{K}\sum_{l=1}^{K}c_{kj}^{\left(i\right)}\phi_{k}(s)\phi_{l}(t).$$ (12)

Let $C_{i}={\left[c_{11}^{\left(i\right)},\ldots ,c_{1K}^{\left(i\right)},c_{21}^{\left(i\right)},\ldots ,c_{2K}^{\left(i\right)},\ldots ,c_{K1}^{\left(i\right)},\ldots ,c_{KK}^{\left(i\right)}\right]}^{T}$ and ϕ(*t*) = [ϕ_1_(*t*),…,ϕ_*k*_(*t*)]^*T*^. Then, Eq (12) can be rewritten as

$$x_{i}(s,t)=C_{i}^{T}(\phi (s)⊗\phi (t)),$$ (13)

where ⊗ denotes the Kronecker product of two matrices.

Define the vector-valued function *X*(*s*, *t*) = [*x*_1_(*s*, *t*), …, *x*_*N*_(*s*, *t*)]^*T*^. The joint expansion of all N random functions can be expressed as

X(s,t)=C(ϕ(s)⊗ϕ(t)) (14)

where the matrix C is given by

$$C={\left[\begin{array}{c} C_{1}^{T}\\ \vdots \\ C_{N}^{T} \end{array}\right]}_{.}$$

In the matrix form, the variance-covariance function of the image signal function can be expressed as

$$\begin{array}{cc} R(s_{1},t_{1},s_{2},t_{2})=\frac{1}{N}X^{T}(s_{1},t_{1})X(s_{2},t_{2})\\ & =\frac{1}{N}[\phi^{T}(s_{1})⊗\phi^{T}(t_{1})C^{T}C[\phi (s_{2})⊗\phi (t_{2})]. \end{array}$$ (15)

Similarly, the eigenfunction β(*s*, *t*) can be expanded as

$$\beta (s,t)=\sum_{j=1}^{K}\sum_{k=1}^{K}b_{jk}\phi_{j}(s)\phi_{k}(t)\;$$

or

$$\beta (s,t)=[\phi^{T}(s)⊗\phi^{T}(t)]b\;,$$ (16)

where *b* = [*b*_11_,…, *b*_1K_,…, *b*_K1_,…, *b*_*KK*_]^*T*^

Substituting expansions Eqs (15) and (16) of the variance-covariance *R*(*s*_1_, *t*_1_, *s*_2_, *t*_2_) and eigenfunction β(*s*, *t*) into the functional eigenequation Eq (9), we obtain

$$[\phi^{T}(s_{1})⊗\phi^{T}(t_{1})]\frac{1}{N}C^{T}Cb=\lambda [\phi^{T}(s_{1})⊗\phi^{T}(t_{1})]b.$$ (17)

Since Eq (17) must hold for all *s* and *t*, we obtain the following eigenequation:

$$\frac{1}{N}C^{T}Cb=\lambda b.$$ (18)

Solving eigenequation Eq (18), we obtain a set of orthonormal eigenvectors *b*_*j*_. A set of orthonormal eigenfunctions is given by

$$\beta_{j}(s,t)=[\phi^{T}(s)⊗\phi^{T}(t)]b_{j},j=1,\ldots ,J.$$ (19)

The random functions *x*_*i*_(*s*, *t*) can be expanded in terms of eigenfunctions as

$$x_{i}(t,s)=\sum_{j=1}^{J}\xi_{ij}\beta_{j}(s,t),i=1,\ldots ,N,$$ (20)

where

$$\xi_{ij}=\int_{S}\int_{T}x_{i}(t,s)\beta_{j}(s,t)dsdt.$$

### Randomized feature selection for *k*—means clustering

The most widely used clustering method in practice is *k*-means algorithm. However, using *k* means to cluster millions or billions of features is not simple and straightforward [11]. An attractive strategy is to select a subset of features and optimize the *k*-means objective function on the low dimensional representation of the original high dimensional data. A natural question is whether the feature selection will lose valuable information by throwing away potentially useful features which could lead to a significantly higher clustering error. Here, we introduce a randomized feature selection algorithm with provable guarantees [6].

For the self-contain, we begin with a linear algebraic formulation of *k*-means algorithm [11]. If we assume there exists a set of *m* points, *A* ^*T*^ = [*P*_1_,…, *P*_*m*_] ∈ *R* ^n×m^, a *k* partition of these *m* points is a collection of *k* non-empty pairwise disjoint sets, *S* = {*S*_1_, *S*_2_,…, *S*_*k*_}, which covers the entire dataset. The objective of *k*-means clustering is to minimize the within-cluster sum of squares. Let *s*_*j*_ = |*S*_*j*_|, be the size of *S*_*j*_. For each *S*_*j*_, its centroid (the mean of data points within the set *S*_*j*_) is defined as:

$$\mu_{j}=\frac{1}{s_{j}}\sum_{p_{i}\in S_{j}}P_{i}$$ (21)

Then *k*-means objective function is written as

$$F(P,S)=\sum_{i=1}^{m}||P_{i}-\mu (P_{i})|{|}_{2}^{2},$$ (22)

where μ(*P*_*i*_) is the centroid of the cluster to which *P*_*i*_ belongs.

The *k*-means objective function can be transformed to a more convenient linear algebraic formulation. A *k*-means clustering *S* of *A* can be represented by its clustering indicator matrix *X* ∈ *R* ^n×m^. Specifically, its element *X*_*ij*_ is defined as

$$X_{ij}=\{\begin{array}{cc} \frac{1}{\sqrt{s_{j}}} & P_{i}\in S_{j}\\ 0 & \text{otherwise.} \end{array}$$ (23)

Each row of *X* has one non-zero element, corresponding to the cluster to which the data point belongs. Each column has *s*_*j*_ non-zero elements, which denotes if the data points belong to cluster *S*_*j*_. The linear algebraic formulation of the *k*-means objective function can be expressed as

$$\begin{array}{l} F(A,X)=||A-XX^{T}A|{|}_{F}^{2}\\ \begin{array}{ccc} & & =\sum_{i=1}^{m}||P_{i}^{T}-X_{i}X^{T}A|{|}_{2}^{2} \end{array}\\ \begin{array}{ccc} & & = \end{array}\sum_{i=1}^{m}||P_{i}^{T}-\mu {\left(P_{i}\right)}^{T}|{|}_{2}^{2}, \end{array}$$ (24)

where $||W|{|}_{F}=\sqrt{Tr(W^{T}W)}$ is the Frobenius norm of a matrix *W*, *X*_*i*_ is the *i*th row of *X*, $X^{T}A={\left[\mu_{1}^{T},\ldots ,\mu_{k}^{T}\right]}^{T}$ and *X*_*i*_ *X* ^*T*^ *A* = *μ*(*P*_*i*_)^*T*^.

Our goal is to find an indicator matrix *X*_*opt*_ which minimizes $||A-XX^{T}A|{|}_{F}^{2}$:

$$X_{opt}=\underset{X\in R^{m\times k}}{\text{argmin}}\;||A-XX^{T}A|{|}_{F}^{2}.$$ (25)

Define

$$F_{opt}=||A-X_{opt}X_{opt}^{T}A|{|}_{F}^{2}.$$ (26)

It is noted that $X_{opt}X_{opt}^{T}A$ has rank at most *k*. The singular value decomposition of the matrix *A* is given by

$$A=U_{k}\Sigma_{k}V_{k}^{T}+U_{\rho -k}\Sigma_{\rho -k}V_{\rho -k}^{T},$$ (27)

where ρ ≤ min(*m*, *n*) is the rank of the matrix *A*. *U*_*k*_ ∈ *R* ^m×k^ and *U*ρ_-*k*_ ∈ *R* ^m×(ρ-k)^ contain the left singular vectors of *A*. *V*_*k*_ ∈ *R* ^n×k^ and *V*_ρ-k_ ∈ *R* ^n×(ρ-k)^ contain the right singular vectors. Singular values σ_1_ ≥ σ_2_ ≥…≥σ_ρ_ > 0 are contained in the matrices Σ_*k*_ ∈ *R* ^k×k^ and Σ_ρ-k_ ∈ *R* ^(ρ-k)(ρ-k)^. Then we can further have $A_{k}=U_{k}\Sigma_{k}V_{k}^{T}=AV_{k}V_{k}^{T}$ and $A_{\rho -k}=U_{\rho -k}\Sigma_{\rho -k}V_{\rho -k}^{T}=A-A_{k}$. Since *A*_*k*_ is the best rank *k* approximation to *A* and $X_{opt}X_{opt}^{T}A$ has rank at most *k*, we obtain

$$||A-A_{k}|{|}_{F}^{2}\leq ||A-X_{opt}X_{opt}^{T}A|{|}_{F}^{2}\leq F_{opt}.$$ (28)

The feature selection for *k*-means clustering algorithm is to select a subset of *r* columns *C* ∈ *R* ^m×r^ from *A*, which is a representation of the *m* data points in the low *r*-dimensional selected feature space. Then, the goal of the *k*-means clustering algorithm in the selected feature space is to find partition of *m* which minimizes $||C-XX^{T}C|{|}_{F}^{2}$:

$${\tilde{X}}_{opt}=\underset{X\in R^{m\times k}}{\text{argmin}}\;||C-XX^{T}C|{|}_{F}^{2}.$$ (29)

Therefore, feature selection is to seek selection of features such that

$$||A-{\tilde{X}}_{opt}{\tilde{X}}_{opt}^{T}A|{|}_{F}^{2}\leq \gamma ||A-X_{opt}X_{opt}^{T}A|{|}_{F}^{2}.$$ (30)

The basic idea of randomized feature selection is that any matrix *C* which can be used to approximate matrix *A* can also be used for dimensionality reduction in the *k*-means cluster analysis [11, 15]. We seek the matrix *C* that minimizes

$$\begin{array}{l} ||A-{\tilde{X}}_{opt}{\tilde{X}}_{opt}^{T}A|{|}_{F}^{2}=||A_{k}-{\tilde{X}}_{opt}{\tilde{X}}_{opt}^{T}A_{k}|{|}_{F}^{2}+||A_{\rho -k}-{\tilde{X}}_{opt}{\tilde{X}}_{opt}^{T}A_{\rho -k}|{|}_{F}^{2}\\ \begin{array}{cccc} & & & \begin{array}{ccc} & & =||AV_{k}V_{k}^{T} \end{array} \end{array}-{\tilde{X}}_{opt}{\tilde{X}}_{opt}^{T}AV_{k}V_{k}^{T}|{|}_{F}^{2}+||A_{\rho -k}-{\tilde{X}}_{opt}{\tilde{X}}_{opt}^{T}A_{\rho -k}|{|}_{F}^{2}\\ \begin{array}{cccc} & & & \begin{array}{ccc} & & =||AV_{k}-{\tilde{X}}_{opt}{\tilde{X}}_{opt}^{T}AV_{k}|{|}_{F}^{2}+||A_{\rho -k}-{\tilde{X}}_{opt}{\tilde{X}}_{opt}^{T}A_{\rho -k}|{|}_{F}^{2} \end{array} \end{array} \end{array}$$ (31)

Let *C* = *AV*_*k*_. Then, $A_{k}=CV_{k}^{T}$. The minimization problem Eq (31) can be reduced to minimizing $||C-XX^{T}C|{|}_{F}^{2}$.

The calculation of the matrix *C* requires the usage of the entire dataset *A*. However, our goal is to select columns of the matrix *A* to approximate *C*. We denote the sampling matrix $\Omega =[e_{i_{1}},\ldots ,e_{i_{r}}]\in R^{n\times r}$, where *e*_*i*_ are the standard basis vectors with its *i*th element being one and all other elements being zeroes. Let *S* ∈ *R* ^r×r^ be a diagonal rescaling matrix. And we further define *C* = *A*Ω*S*. The matrices Ω and *S* can be generated by randomized sampling. Since singular value decomposition of a large matrix *A* may be difficult, we will also use a sampling algorithm to generalize a matrix *Z* which approximates *V*_*k*_. Thus, the matrix *A* can be decomposed to *A* = *AZZ* ^*T*^ + *E*, where the matrix *E* ∈ *R* ^m×n^. We still use ${\tilde{X}}_{opt}$ to denote the output cluster indicator matrix of some γ—approximation matrix on (*C*, *k*). Then, we can estimate the upper bound of the clustering error $||A-{\tilde{X}}_{opt}{\tilde{X}}_{opt}^{T}A|{|}_{F}^{2}$ as follows [6].

$$||A-{\tilde{X}}_{opt}{\tilde{X}}_{opt}^{T}A|{|}_{F}^{2}=||(I_{m}-{\tilde{X}}_{opt}{\tilde{X}}_{opt}^{T})AZZ^{T}+(I_{m}-{\tilde{X}}_{opt}{\tilde{X}}_{opt}^{T})E|{|}_{F}^{2}.$$ (32)

Because *Z* ^*T*^ *E* ^*T*^ = 0_k×m_ we have

$$((I_{m}-{\tilde{X}}_{opt}{\tilde{X}}_{opt}^{T})AZZ^{T}){\left((I_{m}-{\tilde{X}}_{opt}{\tilde{X}}_{opt}^{T})E\right)}^{T}=0_{m\times m}.$$ (33)

Consequently, Eq (32) can be reduced to

$$\begin{array}{l} ||A-{\tilde{X}}_{opt}{\tilde{X}}_{opt}^{T}A|{|}_{F}^{2}=||(I_{m}-{\tilde{X}}_{opt}{\tilde{X}}_{opt}^{T})AZZ^{T}|{|}_{F}^{2}+||(I_{m}-{\tilde{X}}_{opt}{\tilde{X}}_{opt}^{T})E|{|}_{F}^{2}\\ \begin{array}{cccc} & & & \begin{array}{ccc} & & \;\leq \end{array} \end{array}||(I_{m}-{\tilde{X}}_{opt}{\tilde{X}}_{opt}^{T})AZZ^{T}|{|}_{F}^{2}+||E|{|}_{F}^{2} \end{array}$$ (34)

Given Ω and *S*, we have [6]

$$AZZ^{T}=A\Omega S{\left(Z^{T}\Omega S\right)}^{+}Z^{T}+Y$$ (35)

where *Y* ∈ *R* ^m×n^ is a residual matrix and (.)^+^ denotes the pseudo-inverse of a matrix. It is noted that ||*AB*||_*F*_≤||*A*||_*F*_||*B*||_*F*_, $||WZ^{T}|{|}_{F}=\sqrt{\text{Tr}(WZ^{T}ZW)}=||W|{|}_{F}$ and for any two matrices, $||Y_{1}+Y_{2}|{|}_{F}^{2}\leq 2||Y_{1}|{|}_{F}^{2}+2||Y_{2}|{|}_{F}^{2}$.

Then, the first term in Eq (34) can be further bounded by

$$\begin{array}{l} ||(I_{m}-{\tilde{X}}_{opt}{\tilde{X}}_{opt}^{T})AZZ^{T}|{|}_{F}^{2}\leq 2||(I_{m}-{\tilde{X}}_{opt}{\tilde{X}}_{opt}^{T})A\Omega S{\left(Z^{T}\Omega S\right)}^{+}Z^{T}|{|}_{F}^{2}+2||Y|{|}_{F}^{2}\\ \begin{array}{cccc} & & & \begin{array}{cccc} & & & \begin{array}{cc} & \leq 2||( \end{array} \end{array} \end{array}I_{m}-{\tilde{X}}_{opt}{\tilde{X}}_{opt}^{T})A\Omega S|{|}_{F}||(Z^{T}\Omega S|{|}_{F}+2||Y|{|}_{F} \end{array}$$ (36)

Using Eq (30), we obtain

$$\begin{array}{l} ||(I_{m}-{\tilde{X}}_{opt}{\tilde{X}}_{opt}^{T})AZZ^{T}|{|}_{F}\leq 2\gamma ||(I_{m}-X_{opt}X_{opt}^{T})A\Omega S|{|}_{F}^{2}||{\left(Z^{T}\Omega S\right)}^{+}|{|}_{F}^{2}+2||Y|{|}_{F}^{2}\\ \begin{array}{cccc} & & & \begin{array}{cccc} & & & \begin{array}{cc} & \;\leq \end{array} \end{array} \end{array}2\gamma \frac{|(I_{m}-X_{opt}X_{opt}^{T})A\Omega S|{|}_{F}^{2}}{\sigma_{k}^{2}(Z^{T}\Omega S)}+2||Y|{|}_{F}^{2} \end{array}$$ (37)

Since rank (*Z* ^*T*^Ω*S*) = *k*, we have *Z* ^*T*^Ω*S*(*Z* ^*T*^Ω*S*)^+^ = *I*_*k*_ and *AZZ* ^*T*^—*AZZ* ^*T*^Ω*S*(*Z* ^*T*^Ω*S*)^+^ *Z* ^*T*^ = 0_m×n_, which implies that

$$\begin{array}{l} Y=AZZ^{T}-A\Omega S{\left(Z^{T}\Omega S\right)}^{+}Z^{T}\\ \begin{array}{cc} & =AZZ^{T}-AZZ^{T}\Omega S{\left(Z^{T}\Omega S\right)}^{+}Z^{T}-(A-AZZ^{T})\Omega S{\left(Z^{T}\Omega S\right)}^{+}Z^{T} \end{array}\\ \begin{array}{cc} & = \end{array}-(A-AZZ^{T})\Omega S{\left(Z^{T}\Omega S\right)}^{+}Z^{T}. \end{array}$$ (38)

Therefore, we have

$$\begin{array}{l} ||Y|{|}_{F}^{2}=||(A-AZZ^{T})\Omega S{\left(Z^{T}\Omega S\right)}^{+}Z^{T}|{|}_{F}^{2}\\ \begin{array}{cc} & \;\leq || \end{array}(A-AZZ^{T})\Omega S|{|}_{F}^{2}||{\left(Z^{T}\Omega S\right)}^{+}Z^{T}|{|}_{F}^{2}\\ \begin{array}{cc} & \;\leq \end{array}||(A-AZZ^{T})\Omega S|{|}_{F}^{2}||{\left(Z^{T}\Omega S\right)}^{+}|{|}_{F}^{2}\\ \begin{array}{cc} & \;=\frac{||(A-AZZ^{T})\Omega S|{|}_{F}^{2}}{\sigma_{k}^{2}(Z^{T}\Omega S)} \end{array}. \end{array}$$ (39)

Combining Eqs (37) and (39), we obtain:

$$\begin{array}{l} ||(I_{m}-{\tilde{X}}_{opt}{\tilde{X}}_{opt}^{T})AZZ^{T}|{|}_{F}^{2}\leq 2\gamma \frac{|(I_{m}-X_{opt}X_{opt}^{T})A\Omega S|{|}_{F}^{2}}{\sigma_{k}^{2}(Z^{T}\Omega S)}+2||Y|{|}_{F}^{2}\\ \begin{array}{cccc} & & & \begin{array}{cccc} & & & \begin{array}{ccc} & & \leq 2\frac{\gamma |(I_{m}-X_{opt}X_{opt}^{T})A\Omega S|{|}_{F}^{2}+2||(A-AZZ^{T})\Omega S|{|}_{F}^{2}}{\sigma_{k}^{2}(Z^{T}\Omega S)} \end{array} \end{array} \end{array}\\ \begin{array}{cccc} & & & \begin{array}{cccc} & & & \begin{array}{ccc} & & \leq \end{array} \end{array} \end{array}2\frac{\gamma ||(I_{m}-X_{opt}X_{opt}^{T})A\Omega S|{|}_{F}^{2}+||E\Omega S|{|}_{F}^{2}}{\sigma_{k}^{2}(Z^{T}\Omega S)} \end{array}$$ (40)

Combining Eqs (34) and (40) we obtain the following upper bound:

$$||A-{\tilde{X}}_{opt}{\tilde{X}}_{opt}^{T}A|{|}_{F}^{2}\leq 2\frac{\gamma |(I_{m}-X_{opt}X_{opt}^{T})A\Omega S|{|}_{F}^{2}+||E\Omega S|{|}_{F}^{2}}{\sigma_{k}^{2}(Z^{T}\Omega S)}+||E|{|}_{F}^{2}.$$ (41)

The upper bound provide information about how to choose *Z*, Ω and *S*. We chose *Z* to make the residual *E* small. Several terms in the upper bound can be used to guide the selection of the sampling and rescaling matrices Ω and *S*. The first term in the numerator of the upper bound is the clustering error of the input partition in the reduced dimension space. We chose Ω and *S* to make this clustering error small. The residual *E* is involved in the second term of the numerator and final term in the inequality Eq (41). We chose Ω and *S* such that they will not substantially increase the size of the residual *E*. The term in the denominator involves *Z*,Ω and *S*. Therefore, the selected Ω and *S* do not significantly change the singular structure of the projection matrix *Z* and ensure that $\sigma_{k}^{2}(Z^{T}\Omega S)$ is large. Under these guidances, the following randomized feature selection algorithm can be developed.

### Randomized feature selection algorithms

Let *k* be the number of clusters and ε be the errors that are allowed. Set $r=k+\left[\frac{k}{\varepsilon }+1\right]$ as the number of features being selected [16]. Consider data matrix $A=\left[\begin{array}{ccc} a_{11} & \cdots & a_{1n}\\ \vdots & \cdots & \vdots \\ a_{m1} & \cdots & a_{mn} \end{array}\right]$. Let *i* denote the index of the individual sample and *j* be the index of feature. We intend to select *r* features.

Procedures of algorithms are given as follows.

1. Generate an *n*×*r* standard Gaussian matrix G, with *G*_*ij*_ ~ *N*(0,1).

2. Let *Y* = *AR* ∈ *R* ^m×r^.

3. Orthonormalize the columns of the matrix *Y*, which leads to the matrix *Q* ∈ *R* ^m×r^.

4. Singular value decomposition of the matrix *Q* ^*T*^ *A*: *Q* ^*T*^ *A = U*Σ*V* ^*T*^.

Let *Z* ∈ *R* ^n×k^ be the top *k* right singular vectors of *Q* ^*T*^ *A*, i.e., *Z* = [*V*_1_,…, *V*_*k*_].

5. Calculate the sampling probability:

qi=||Z(i)||22||Z||F2,i=1,…,n,∑i=1nPRi=1, where *Z*_(i)_ is the *i*-th row of the matrix *Z* and $\left||Z|{|}_{F}^{2}=tr(ZZ^{T}\right)$.

6. Initiate Ω = 0_n×r_ and *S* = 0_r×r_.

For *t* = 1,…, *r*, pick an integer *i*_*t*_ from the set {1,2,…, *n*} with probability $q_{i_{t}}$ and replacement, set Ω(*i*_*t*_, *t*) = 1 and $S(t,t)=\frac{1}{\sqrt{rq_{i_{t}}}}$.

End

7. Return *C* = *A*Ω*S* ∈ *R* ^m×r^.

## Results

We tested our algorithm on two distinct cancer histology image datasets downloaded from the TCGA database (https://tcgadata.nci.nih.gov/tcgafiles/ftp_auth/distro_ftpusers/anonymous/tumor/ov/bcr/intgen.org/diagnostic_images/ and https://tcgadata.nci.nih.gov/tcgafiles/ftp_auth/distro_ftpusers/anonymous/tumor/kirc/bcr/intgen.org/diagnostic_images/). The first dataset is an ovarian cancer dataset, which includes 176 histology images taken from 106 drug sensitive and 70 drug resistant tissue samples. The second dataset is a kidney cancer histology dataset which includes 188 histology images. 121 of these histology images are taken from kidney renal clear cell carcinoma (KIRC) samples and the rest of them are from the normal samples.

We compared the performance of our algorithm with the standard *k*-means and regularization-based sparse *k*-means clustering algorithms [8]. We also compared the performance of the two dimensional FPCs with the Fourier expansions and SIFT descriptors. We use the clustering accuracy (ACC) which is defined as the proportion of correctly clustered images, clustering sensitivity which is defined as the proportion of correctly clustered drug sensitive or tumor samples, and clustering specificity which is defined as the proportion of correctly clustered drug resistant or normal samples, for performance evaluation in this study.

### Comparison of two dimensional FPCA with Fourier expansion and SIFT descriptor

**Fig 1:**
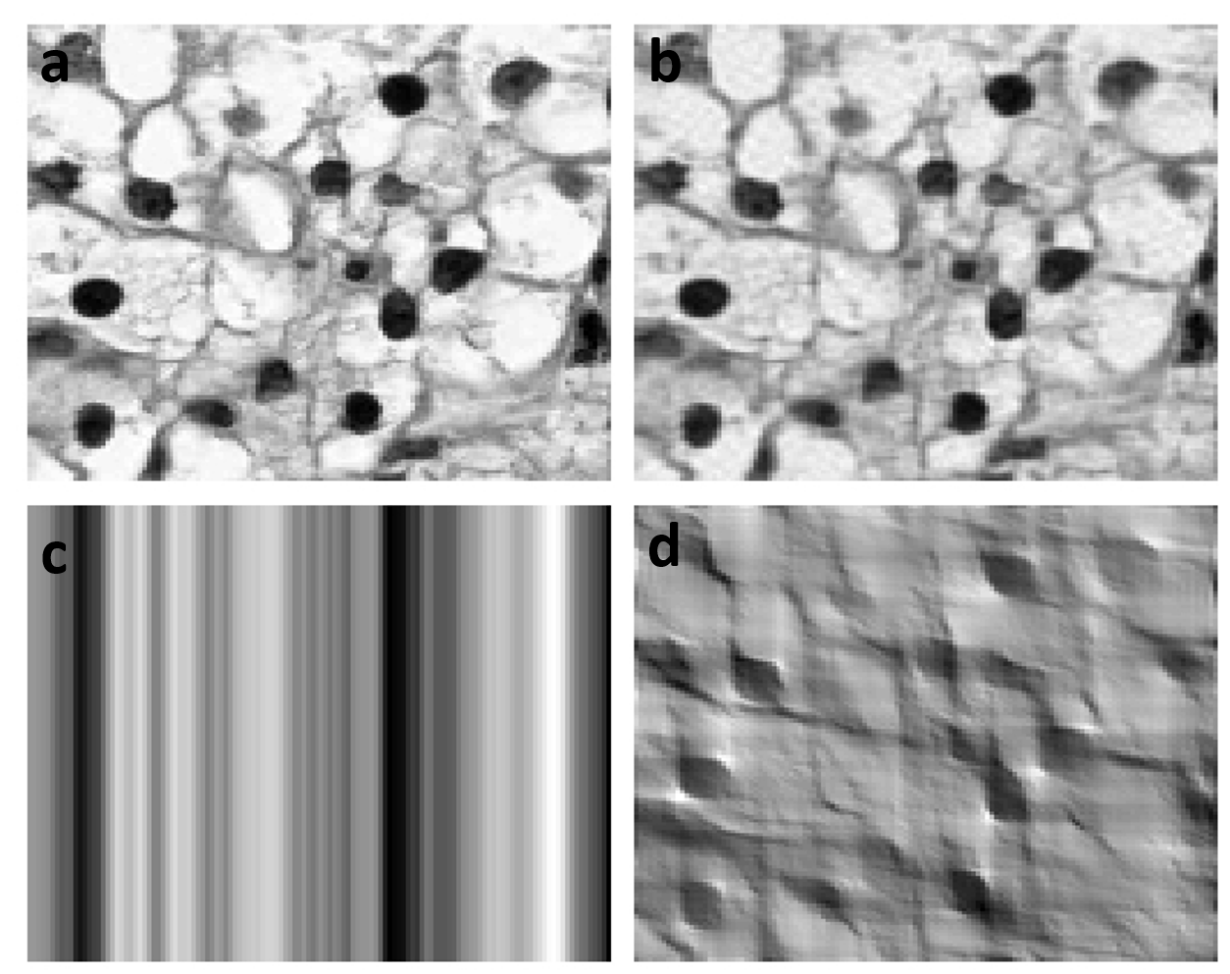
(a) Original image of one of the 121 histology images of the kidney cancer cells which were downloaded from the TCGA database, (b) reconstruction of the original histology images of kidney cancer cells by using its 133 FPCA scores, (c) reconstruction of the original kidney histology image by using its first 133 Fourier expansion coefficients, (d) reconstruction of the original kidney histology image by using its first 4,357 Fourier expansion coefficients.

To intuitively illustrate the power of FPCs in the dimension reduction of image data, we first presented Fig 1 which showed the original and reconstructed the KIRC tumor cell images. We observed that the reconstructed the KIRC tumor cell images using only 133 FPCs are very close to the original images. However, even when we used the 4,357 terms in the Fourier expansion to reconstruct KIRC cell images, the reconstructed images were still unclear. Then, we compared the accuracies of the standard k-means algorithms and randomized sparse k-means algorithms for clustering ovarian cancer and KIRC tissue samples using FPC scores (188 components), Fourier expansion coefficients (65025 components), SIFT descriptors, GPCA (http://cran.r-project.org/web/packages/sGPCA/index.html), MPCA (http://cran.r-project.org/web/packages/rTensor/index.html) as image features. The results were summarized in Table 1. From Table 1 we can see that the cluster analysis using FPC scores as features has a higher accuracy than using Fourier expansion coefficients, SIFT descriptors, GPCA and MPCA image feature extraction for both the standard k-means and randomized sparse k-means and both the ovarian cancer and KIRC datasets.

**Table 1:** Performance of standard and randomized sparse k-means clustering algorithm for FPCA, MPCA, GPCA, SIFT and Fourier expansion.

Methods	Feature	Ovarian Cancer			KIRC		
	Extraction	Accuracy	Sensitivity	Specificity	Accuracy	Sensitivity	Specificity
Standard	FPCA	0.570	0.660	0.400	0.809	0.917	0.612
k-means	MPCA	0.529	0.538	0.522	0.803	0.901	0.627
	GPCA	0.522	0.519	0.529	0.787	0.901	0.582
	SIFT	0.557	0.547	0.547	0.681	0.587	0.701
	Fourier	0.557	0.557	0.557	0.803	0.917	0.597
Randomized	FPCA	0.653	0.793	0.486	0.835	0.926	0.672
sparse	MPCA	0.539	0.538	0.543	0.819	0.918	0.642
k-means	GPCA	0.527	0.538	0.507	0.803	0.918	0.597
	SIFT	0.608	0.708	0.457	0.729	0.818	0.567
	Fourier	0.608	0.679	0.500	0.814	0.884	0.687

### Performance of standard k-means clustering algorithm, sparse k-means clustering algorithm and randomized sparse k-means clustering algorithm

We compared the performance of the standard *k*-means clustering algorithm, the sparse *k*-means clustering algorithm and randomized sparse *k*-means clustering algorithm in both the ovarian and KIRC cancer studies. The “SPARCL” package was used for implementing the sparse k-means clustering algorithm [8]. The SIFT descriptor [17] was used as another tool for image representation. The images in the ovarian cancer study were taken before any treatment. Therefore, the images were used to predict the drug response. The results were summarized in Table 2. Table 2 showed that the randomized k-means clustering algorithms used significantly fewer features, but achieved higher accuracy than both the standard *k*-means and sparse *k*-means algorithms.

**Table 2:** Performance of standard K-means, sparse K-means and randomized K-mean clustering algorithm using the SIFT descriptor clustering algorithm using the SIFT descriptor.

	Ovarian Cancer				KIRC			
	Features	Accuracy	Sensitivity	Specificity	Features	Accuracy	Sensitivity	Specificity
K-means	2,560	0.547	0.547	0.547	2,560	0.681	0.587	0.701
Sparse K-means	574	0.545	0.472	0.657	597	0.585	0.62	0.522
Randomized K-means	70	0.608	0.708	0.457	100	0.729	0.818	0.567

### Performance of standard k-means, sparse k-means and randomized sparse k-means clustering algorithms using FPC scores

We studied the performance of standard k-means, sparse k-means and randomized sparse k-means clustering algorithm using the FPC scores as the image features. The results of the performance for different clustering algorithms to the two cancer imaging datasets were summarized in Table 3. Again, the randomized sparse k-means algorithms used the smallest number of FPC scores, but had the highest clustering accuracy, followed by sparse k-means clustering algorithms. The standard k-means clustering algorithms used the largest number of FPC scores, but achieved the lowest clustering accuracy. Comparing Table 3 with Table 2, we found that FPCA substantially improved clustering accuracy. Specifically, for the KIRC dataset we observed that replacing the SIFT descriptor with FPC scores increased the clustering accuracies of the stand k-means, sparse k-means and randomized sparse k-means from 68.09% to 80.85%, 58.51% to 81.91%, and 72.87% to 83.51%, respectively.

**Table 3:** Performance of standard k-means, sparse k-means and randomized sparse k-means clustering algorithms using FPC scores.

	Ovarian Cancer				KIRC			
	Features	Accuracy	Sensitivity	Specificity	Features	Accuracy	Sensitivity	Specificity
K-means	176	0.574	0.660	0.400	188	0.809	0.917	0.612
Sparse K-means	81	0.585	0.670	0.457	92	0.819	0.819	0.642
Randomized sparse K-means	23	0.653	0.793	0.486	5	0.835	0.926	0.672

### Performance of standard spectral, sparse K-means, and randomized sparse spectral clustering algorithms using Fourier expansion coefficients

To further evaluate the performance of randomized sparse clustering algorithm, we used three algorithms: standard spectral, sparse k-means and randomized spectral clustering algorithms with Fourier expansion coefficients to conduct clustering analysis for the ovarian cancer and KIRC datasets. Table 4 was presented to summarize the results. The performances of the three clustering algorithms using Fourier expansion coefficients as imaging features were the same as that using other features. Sparse algorithms will improve cluster accuracy and randomized sparse clustering algorithms had the highest accuracy among the three clustering algorithms. We also observed that in general, using Fourier expansion coefficients as imaging features had less accuracy than using FPC scores as features.

**Table 4:** Performance of standard spectral, sparse K-means clustering and sparse spectral with randomized feature selection clustering algorithms with Fourier expansion.

	Ovarian Cancer				KIRC			
	Features	Accuracy	Sensitivity	Specificity	Features	Accuracy	Sensitivity	Specificity
Spectral clustering	65025	0.557	0.557	0.557	65025	0.803	0.917	0.597
Sparse K-means	959	0.545	0.500	0.614	161	0.819	0.917	0.642
Randomized Spectral clustering	100	0.642	0.576	0.743	10	0.835	0.926	0.672

### Multiple cluster analysis

**Fig 2:**
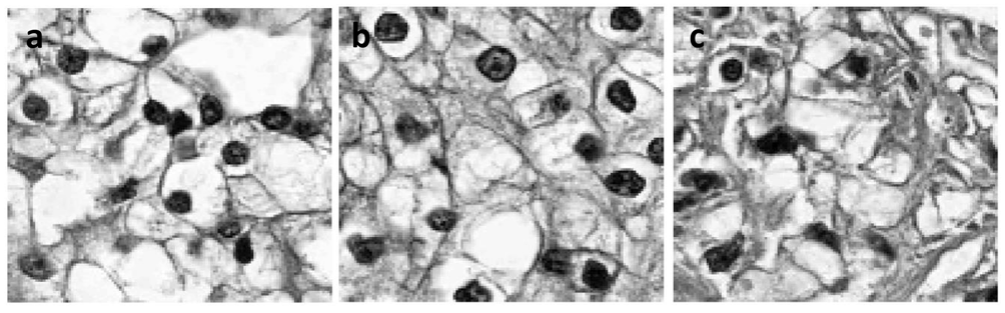
Historic pathology images. (a) Pathology grades 1 and 2, (b) pathology grade 3 and (c) pathology grade.

Generally, a population can be divided into two groups: normal and patient groups. However, the patients’ subpopulation is highly heterogeneous and has complex structures. Patients need to be further divided into several more homogeneous groups. Table 5 presented results of three clustering algorithms for multiple cluster analysis in the KIRC studies where tumor cells were partitioned into three groups. Neoplasm histologic grade which is based on the microscopic morphology of a neoplasm with hematoxylin and eosin (H&E) staining (G1, G2, G3 and G4) was selected as the prognostic factors of survival [18]. In the present analysis, the patients of G1 and G2 were regrouped as group 1 patients. Patients of G3 were regrouped as group 2 patients and patients of G4 were regrouped as group 3 patients. Table 5 suggested that the randomized sparse k-means had the highest accuracy for clustering KIRC tumor cell grades, followed by sparse k-means and standard k-means clustering algorithms, where the accuracy was defined as the proportion of individuals who were correctly assigned to the groups. As shown in Fig 2, clustering tumor cells has a close relationship with cell pathology which characterizes progressing and development of tumors. In Fig 2a, morphology of nucleus that was represented by black circles changed slowly. When disease proceeds nucleus became large and expanded (Fig 2b). When tumors proceeded to the final stage, the nucleus was metastated and became blur (Fig 2c).

**Table 5:** Performance of standard k-means, sparse k-means and randomized k-means algorithms for clustering KIRC tumor cell grades.

		TRUE		
Method	Assigned	Group1	Group 2	Group 3
	Group 1	17 (58.6%)	15 (53.6%)	7 (50.0%)
K-means	Group 2	12 (41.4%)	12 (42.9%)	7 (50.0%)
	Group 3	0	1 (3.4%)	0
	Accuracy	40.80%		
	Group 1	10 (34.5%)	6 (21.4%)	3 (21.4%)
	Group 2	13 (44.8%)	17 (60.7%)	7(50.0%)
Sparse K-means	Group 3	6 (20.7%)	5 (17.9%)	4 (28.6%)
	Accuracy	43.70%		
	Group 1	14 (48.3%)	4 (14.3%)	2 (14.3%)
Randomized sparse K-means	Group 2	8 (27.6%)	20 (71.4%)	8 (57.1%)
	Group 3	7 (24.1%)	4 (14.3%)	4 (28.6%)
	Accuracy	53.50%		

### Robustness of the proposed random sparse k-means clustering algorithms

To evaluate the robustness of the proposed sparse k-means for clustering analysis, we performed simulations. We repeated the randomized sparse k-means clustering for 100 times using two dimensional FPC and the KIRC data. We selected 5 features from a total of 188 FPC features each simulation. The results were summarized in Tables 6 and 7. We observed that the same 5 features were selected in more than 94% of simulations and we can reach 83.5% clustering accuracy in more than 93% of simulations. The results showed that the proposed random sparse k-mean clustering methods were very stable.

**Table 6:** Percentage of the simulations sharing the same FPC features in KIRC study.

Number of Features	2	1	2	1	1	1	1
Percentage of simulation sharing same features	100%	96%	94%	7%	5%	3%	1%

**Table 7:** Stability of the estimated accuracy using the randomized sparse k-means clustering and FPC in KIRC study.

Percentage	Accuracy	Sensitivity	Specificity
93%	0.835	0.926	0.672
6%	0.824	0.909	0.672
1%	0.819	0.917	0.642

## Discussion

In this paper, we proposed to combine feature extraction and feature selection for cluster analysis of the imaging data and developed FPCA-based randomized sparse clustering algorithms. Because the image data are always of high dimension, the dimension reduction is a key to the success of imaging cluster analysis. To successfully perform image cluster analysis, we addressed several issues for dimensional reduction in the sparse image cluster analysis.

The first issue we addressed is the applications of the feature extraction technique to the image data dimension reduction. In other words, we construct a small set of new artificial features that are often linear combinations of the original features and then the *k*-means method is used to cluster the constructed features. A variety of methods for feature extraction has been developed such as PCA or FPCA. However, FPCA is developed for one dimensional data and cannot be simply applied to two or three dimensional imaging data. Here we extended FPCA from one dimension to two or three dimensions and applied it to extract the features from image data. Real histology imaging cluster analysis showed that the FPCA for imaging dimension reduction substantially outperformed the SIFT descriptor and Fourier.

The second issue is to develop a sparse clustering algorithm which attempts to identify the features underlying the clusters and remove noise and the irrelevant variables. Generally, there are two types of sparse clustering algorithms. One type of the algorithms is to optimize weighted within-cluster sum of squares by using the lasso type penalty to select the weights and the features. The difficulty with this type of constrained based sparse clustering algorithms is the determination of the threshold which is used to remove the redundant features. In theory, the features with non-zero weights are selected for clustering analysis. However in practice, all the weights vary continuously. The determination of an appropriate threshold to cut off the irrelevant features is a big challenge. An alternative approach is to randomly and directly select a small subset of the actual features which can ensure to approximately reach the optimal *k*-means objective value. Both mathematical formulations of the *k*-means objective function and sampling algorithms to optimize objective function have well been developed. We can expect that the randomized sparse *k*-means clustering algorithms can work very well. By applying the sparse clustering algorithms to the real cancer histology image data, we showed that both randomized *k*-means clustering and lasso-type k-means clustering algorithms substantially outperformed the standard k-means algorithm, and the performance of the randomized k-means sparse clustering algorithm was better than that of the lasso type sparse k-means clustering algorithms.

The third issue is to combine feature extraction and feature selection. Feature extraction and feature selection are two major tools for dimension reduction. In imaging cluster analysis, feature extraction and feature selection are often used separately for data reduction. The main strength of our approach is to integrate feature extraction and feature selection into a dimension reduction tool before clustering the images. We first performed two dimensional FPCA of images as a feature extraction tool to extract group structure information of the images. The resulting vectors of FPC scores which contain image group information were used to represent the features of the images. Then, we designed a random matrix column selection algorithm to select some components of the vector of FPC scores for further cluster analysis. Finally, the *k*-means method was used to cluster the selected FPC scores. We showed that *k*-means method with feature extraction and feature selection as dimension reduction had the highest cluster accuracy in two real cancer histology images clustering studies.

Appropriate usage of feature extraction and feature reduction may substantially improve the performance of clustering algorithms. This conclusion does not depend on which clustering algorithms are selected. We demonstrated that cluster accuracies of both sparse *k*-means and sparse spectral clustering were higher than standard *k*-means and spectral clustering.

The proposed method provides a powerful approach to image cluster analysis, but some challenges still remain. The randomized feature selection algorithms have deep connections with the objective function of k-means clustering and low-rank approximations of the data matrix. However, the solutions to optimize the objective function of *k*-means clustering may not correspond to the true group structure of the image data well. The selection of the number of features also depends on the accuracy of low-rank approximation. Although we can provide theoretic calculation of the number of selected features, in practice we need to automatically calculate it by iterating the feature selection algorithm from the data, which requires heavy computation for large datasets. The randomized feature selection for multiple groups clustering still has serious limitation. Clustering images into multiple groups is an important, but a challenge problem. The main purpose of this paper is to stimulate the discussion about what are the optimal strategies for high dimensional image cluster analysis. We hope that our results will greatly increase confidence in applying the dimension reduction to image cluster analysis.

## Conclusions

We extended one dimensional FPCA to the two dimensional FPCA and develop novel sparse cluster analysis methods which combine two dimensional FPCA with randomized feature selection to reduce the high dimension of imaging data. We used stochastic calculus to derive the formula for the calculation of the variance of integral of weighted linear combination of two dimensional signals of the images. We formulated two dimensional FPCA as a maximization of this variance with respect to weight function (functional components) of two variants and used variation of theory to find solutions that are the solutions to the integral equations with two variants. We used functional expansion to develop computational methods for solving integral equations with respect to functional components and finding FPC scores which are taken as features for cluster analysis.

Followed the approach of [6] we explored matrix approximation theory and a technique of [19] to design a randomized method to select FPC scores as features for cluster analysis with probability that are correlated with the right singular vectors of the FPC score matrix. In theory, we can prove that the randomized feature selection algorithm guarantees the quality of the resulting clusters. The developed randomized algorithms which integrate FPC scores as features for dimension reduction can be applied to *k*-means and spectral clustering algorithms. Results on clustering histology images in the ovarian cancer and KIRC cancer studies showed that the randomized *k*-means and spectral clustering algorithms integrating FPCA substantially outperform other existing clustering algorithms with and without feature selections. The randomized sparse clustering algorithms integrating FPCA is a choice of methods for image clustering analysis.

## Supporting Information

S1 Table The computational cost of the standard k-means and randomized sparse k-means clustering algorithms using four feature extraction methods. (XLSX)
